# Temporomandibular Disorders: Knowledge, Attitude and Practice among Dentists in Tehran, Iran

**DOI:** 10.5681/joddd.2010.023

**Published:** 2010-09-16

**Authors:** Maryam Baharvand, Majid Sedaghat Monfared, Mina Hamian, Elnaz Jalali Moghaddam, Fatemeh Sadat Hosseini, Kaveh Alavi Alavi

**Affiliations:** ^1^ Associate Professor, Department of Oral Medicine, Faculty of Dentistry, Shaheed Beheshti University of Medical Sciences, Tehran, Iran; ^2^ Assistant Professor, Department of Prosthodontics, Faculty of Dentistry, Shaheed Beheshti University of Medical Sciences, Tehran, Iran; ^3^ Resident of Oral Medicine, Faculty of Dentistry, Shaheed Beheshti University of Medical Sciences, Tehran, Iran; ^4^ Dentistry Student, Faculty of Dentistry, Shaheed Beheshti University of Medical Sciences, Tehran, Iran; ^5^ General Dental Practitioner, Tehran, Iran; ^6^ MD, Consultant of Biostatistics, Faculty of Medicine, Iran University of Medical Sciences, Tehran, Iran

**Keywords:** Attitude, dentist, knowledge, practice, temporomandibular disorders

## Abstract

**Background and aims:**

Temporomandibular disorders (TMD) account for the most common orofacial pains rising from musculoskeletal origin. The aim of this study was to investigate the level of knowledge, attitudes and practice of dental practitioners regarding TMD in Tehran, Iran.

**Materials and methods:**

A questionnaire, containing 29 questions on etiology, signs and symptoms, diagnosis and treatment of TMD, was given to 200 randomly selected general dental practitioners and specialists as well as 11 TMD ex-perts.

**Results:**

An overall response rate of 97% was achieved among participants (mean age: 39 ± 8.2 years old; mean years in practice: 11.5 ± 7.4). The mean score of knowledge of TMD was found to be 10.85± 2.54 (of a total of 23). TMD specialists were significantly more knowledgeable than general dental practitioners (p<0.05). With respect to attitude, there was a significant difference among various age groups, and by increasing age and years in practice, the attitude towards TMD had weakened. However, no significant difference was recorded between general dental practitioners’ attitude and that of TMD experts towards TMD. There was a positive correlation between subjects’ knowledge and attitude (P= 0.007, r=0.138).

**Conclusion:**

According to the results, the level of knowledge and attitude of general dental practitioners of Tehran regarding TMD is not desirable. The majority are not willing to admit and treat TMD patients.

## Introduction

Temporomandibular disorder (TMD) is a complex, multifaceted disorder characterized by pain and tenderness in the temporomandibular joints, mastication muscles, and adjacent soft tissues. Limitation in mouth opening or mandibular lateral movements or protrusion as well as the associated sounds in temporomandibular joint are also associated with TMD.^1 - 3^ Generally, TMD accounts for the most common orofacial pains rising from musculoskeletal origin.^4^ The diagnosis of orofacial pain can be quite difficult and often presents a serious challenge to most practitioners. Having sufficient knowledge and skill regarding TMD helps the dentist in recognizing any irregularities in the temporomandibular joint during examination, and then, referring the patient to the appropriate professionals, thereby terminating the cycle of perpetual medical referrals. ^5 - 7^

To assess the knowledge and beliefs of general dental practitioners regarding TMD, several studies have been carried out in different countries. In one study, there was a high degree of discrepancy in TMD etiology, diagnosis, and treatment between general dental practitioners and TMD specialists, which was an indication of dentists’ lack of knowledge and up-to-date information regarding TMD.^8^ In another study, the participating dentists generally agreed with TMD experts in the ‘etiology’ domain, but disagreed with experts in domains of ‘pathophysiology, diagnosis, and treatment’ and had insufficient knowledge concerning these areas.^9^ Similar findings were seen in other studies.^10 , 11^ In general, TMD patients were not welcomed by practicing dentists in Seoul and they were usually referred to the orofacial pain clinics of universities.^11^

However, it has been shown that a general dental practitioner could individually predict treatment outcome with similar results as a TMD specialist in selected patients diagnosed with TMD.^5^ In fact, a high degree of agreement in knowledge between Swedish general dental practitioners and TMD specialists has been shown.^6^ Another study has revealed an acceptable level of knowledge of TMD amongst Iranian general dental practitioners, which indicates the issue has not been neglected. The relation between dentists’ knowledge and attitude, however, has not yet been studied. The aim of the present study was to evaluate the knowledge of TMD and chronic pain in four domains of etiology, signs and symptoms, diagnosis, and treatment among a group of Iranian dentists. Furthermore, the correlation of the dentists’ level of knowledge and attitude regarding TMD was assessed.

## Materials and methods

This cross-sectional study was designed to include the dentists practicing in dental clinics and private offices of Tehran, Iran. After obtaining a list of names and addresses from Iranian Dental Association, 200 dentists including ‘general dental practitioners’ and ‘specialists’ were selected randomly. A questionnaire consisting of 29 queries about TMD, chosen from a relevant textbook,^1^ was handed to each participant by referring to their office or clinic. All participants were informed of the aim of the study, and were assured of confidentiality of their answers. The subjects were asked to complete the survey within one week. The questionnaire was also given to 11 ‘TMD experts’ chosen from the academic staff at Shaheed Beheshti University of Medical Sciences. Considering the fact that they are more involved in management of patients with TMD than other dentists, their answers were considered as standard for comparison with other participants. The questionnaire consisted of three main sections: knowledge, attitude, and practice. The section on knowledge consisted of 18 triple-choice questions (agree, disagree, indifferent), and 1 multiple-choice question, covering four domains of etiology, sign and symptoms, diagnosis, and treatment. The section on attitude consisted of 5 multiple-choice and yes/no questions in a case presentation form, and the part concerning practice contained 5 multiple-choice questions regarding the respondents’ opinion towards acceptance of TMD patients as well as acquiring sufficient knowledge of TMD and the approach of each practitioner toward this issue. To ensure reliability of the survey, a pilot study was performed, in which the questionnaires were handed to 30 other practicing dentists outside the city. This was repeated two times with a 10-day interval. The reliability was evaluated based on agreement of the answers in two trials using kappa coefficient and Spearman’s correlation coefficient (p = 0.016, r = 0.462).

Questionnaires were collected after one week. In cases of lost or incomplete questionnaires, a reminder was sent, up to 3 times, and if such participants were not willing to cooperate they were excluded from the study. After collecting the completed questionnaires, they were graded a positive mark for every correct answer and zero for every incorrect answer. The final score was calculated by summing up the positive marks for each respondent. In the evaluation of the knowledge section, the following classification was used:

- score of 1- 6: low level of knowledge
- score of 7- 12: relatively low level of knowledge
- score of 13- 18: fair level of knowledge
- score of 19 and up: high level of knowledge^1^

In the evaluation of attitude, the following classification was used:

- score of 0- 2: weak
- score of 3- 4: fair
- score of 5.0: good

Data were presented with descriptive statistics. To investigate the relationship between knowledge and attitude of different groups, chi-square test was used. Pearson’s correlation coefficient was used to evaluate the relation amongst the quantitative components. All data were analyzed using SPSS 11.5 computer software. A p value < 0.05 was considered statistically significant.

## Results

The overall response rate of participants was 97%. Seven general dental practitioners were excluded from the study. From among 193 investigated dentists (135 men and 58 women; mean age: 39 ± 8.2 years old; mean years in practice: 11.5 ± 7.4), 178 participants were general dental practitioners and 15 were dental specialists. Comparison between factors influencing attitude as well knowledge and practice are shown in Tables 1 & 2.

**Table 1:** Comparison between factors influencing attitude of dentists and experts regarding temporomandibular disorders

Factors influencing attitude						
Investigating attitude	Gender	Age	Number of years in practice	Being a specialist	Location of education	Level of awareness
Treatment in private office	NS	S	NS	S	NS	S
Training during education	NS	NS	NS		S	NS
Attending continuing education courses			NS	NS		NS

S: significant (p < 0.05); NS: not significant.

**Table 2:** Comparison of effective factors on knowledge and practice of temporomandibular disorders among dentists and experts

Effective factors	Knowledge	Practice
Gender	NS (p =0.3)	NS (p = 0.8)
Age	NS (p = 0.3)	S (p = 0.02)
Number of years at practice	NS (p = 0.14)	S (p = 0.03)
Being a specialist	S (p = 0.01)	S (p = 0.03)
University of education	S (p = 0.01)	S (p = 0.02)
Subscription to dental journals	S (p = 0)	S (p = 0.01)
Attending continuing education courses	S (p = 0)	NS (p = 0.69)

S: significant (p < 0.05); NS: not significant.

Mean knowledge score of all participants was 10.85± 2.54 (of a total of 23 achievable scores). 3% of dentists were rated as having a low level of TMD knowledge, 72% were rated as having a relatively low level of TMD knowledge, and 25% were rated as having a fair level of knowledge towards TMD. None of the dentists were recognized as having a high level of TMD knowledge. The mean score of TMD experts was 14.27. There was a statistically significant difference in TMD knowledge between general dental practitioners and TMD experts (p= 0.016).

In the treatment domain, TMD specialists and general practitioners showed an agreement in their responses. However, the two groups were in disagreement regarding the domains of etiology, diagnosis, and signs and symptoms (p= 0.013). The TMD experts were more knowledgeable in these areas.

All dentists with 30 years at practice or more were classified as having low level in knowledge of TMD. There was a significant difference in knowledge of TMD among different age groups (p= 0.030). The knowledge level was relatively low in the age group of 55 years old and older. The same trend was observed with the attitude level.

The mean attitude scores (total = 5) of practicing dentists and TMD experts were found to be 3.1, and 3.54, respectively. 21% of dentists were rated as having a weak attitude towards TMD patients, 77% were rated as having a fair attitude towards TMD patients, and 2% were classified as having a good attitude towards these patients. There was a significant difference in the attitude among various age groups (p=0.039); by increasing age and years at practice, the attitude towards TMD was weakening.

Differences in knowledge and attitude between male and female dentists were not significant.

The correlation between the level of knowledge dentists and tendency to accept TMD patients was significant (p= 0.019) in the number of TMD patients being visited by the more knowledgeable dentists. These patients were mostly seen by the group of TMD experts, especially prosthodontists.

Only a small number of the dentists in all 3 groups (general dentists, specialists, and experts) held the same opinion regarding the adequacy of undergraduate dental education in TMD and orofacial pain. The same results were obtained regarding the continuous education programs towards TMD.

In response to the last question of the attitude section, asking the subjects about the sources they were willing to gain relevant knowledge of TMD, the experts were mostly willing to use the internet. Also, with respect to referral to the reference books, the groups of specialists and experts showed the greatest tendency compared to the general practitioners. 4.7% of the respondents were relying heavily on their personal experiences.

## Discussion

According to the results of the present study, the majority of the dentists have a low level of knowledge towards temporomandibular disorders. Considering the mean attitude score of 3.1 (of a total of 5), it is obvious that the majority of the respondents are in a fair status of attitude towards TMD and about the practice.

In comparison, specialists showed a higher level of knowledge towards TMD than general dentists. Nearly 80% of specialists considered reference books as their source of obtaining information regarding TMD. In addition, one-third of specialists regarded the internet as their primary source of information. This can be the reason for the higher level of knowledge among specialists, since the information gained from the internet can be more up-to-date than that acquired from the reference books.

This study evaluated the knowledge of dentists towards TMD in four domains of etiology, signs and symptoms, diagnosis, and treatment. The lowest level of knowledge amongst the dentists was shown to be in the etiology domain (93.8% of respondents). A fair level of knowledge was only seen in the domain of signs and symptoms. This can be explained by the fact that during the undergraduate years, the majority of dentists mostly receive courses focusing on the signs and symptoms, diagnosis, and treatment of TMD, paying less attention to the underlying etiology. Furthermore, lack of encounter with TMD patients by dental students during their education has led to s lack of practice and knowledge regarding the etiologic factors. The same trend is present even during their practice when confronting a TMD case, where the dentist does not put enough effort into looking for the underlying cause and makes a hasty diagnosis on the sight of the first sign and symptom, leading to a treatment that only relieves the pain and discomfort in the least amount of time possible. The fact of relying on personal experiences is also confirmed by this theory which is an indication of the dentists’ weak ability to figure out the underlying etiology.

Reaching a consensus on treatment issues between general practicing dentists and TMD experts maybe related to the limited number of treatments regarding temporomandibular disorders. Further evaluation of treatment options in detail will reveal the differences in the knowledge domain. In other words the treatment phases need a higher level of knowledge than treatment appellation. In the study of Just al,^8^ there was a high degree of discrepancy about TMD etiology, diagnosis, and treatment between general practitioners and TMD specialists. These results are almost in accordance with those of this study. The results of Resche et al,^9^ however, do not concord with those of the current study. In the latter study, the participating dentists generally had more knowledge in the etiology domain, but concerning pathophysiology, diagnosis, and treatment domains, their level of knowledge was weak and insufficient. However, similar to the findings of this study, regarding knowledge and attitude, differences in knowledge between male and female dentists were not significant, while a considerable difference in opinions was found between general practicing dentists and specialists. Contrary to the results of the current study, there was no considerable discrepancy between views of practicing dentists and those of TMD experts in a previous study, and it was concluded that the participating dentists had more knowledge regarding etiologic factors, while in domains of pathophysiology, diagnosis, and treatment, their lack of knowledge was obvious.^10^

The findings of the present study partially replicate the results of another study.^11^ Although in that study, the participating dentists showed a desirable level of knowledge regarding etiologic factors of TMD, rarely were they willing to visit TMD patients.^11^

There was a high consensus in TMD knowledge among the specialists and a high degree of agreement found between general practitioners and TMD specialists in another study assessing the dentists’ knowledge of TMJ disorders in children and adolescents.^5^ In some areas, however, TMD specialists still needed to reach a consensus. TMD specialists differed most in opinion in the domain diagnostic and classification.^5^ In 65% of statements, differences in knowledge between general practitioners and TMD specialists were non-significant; but, in the domain of treatment and prognosis, the differences were significant. Most of these statements were related to morphological factors.^5^

The results of a similar study regarding dentists’ knowledge of TMD in Iran showed that general dental practitioners’ knowledge of TMD and its related factors was good and acceptable.^12^ This finding is not in line with the results of our study. This difference may be due to the used questionnaire, which contains only questions regarding knowledge, not attitude and the practice, and the investigated group, which is only general practitioners and does not conclude experts and specialists: 400 general dentists were investigated. 13 % were categorized as having a low level of knowledge while 50% had good and excellent level of knowledge of TMD. 10% had low a level of awareness of TMD, and more than 60% were completely familiar with related factors.^12^

Compared to practicing dentists, the TMD experts in this study had a higher level of knowledge of TMD in etiology, signs and symptoms, and diagnosis domains. However, in the treatment domain, the experts are almost in agreement with the practicing dentists, which again can lead to the conclusion that the presumed ability to present a correct and effective treatment is not high enough among respondents. Rapidly-growing concepts on temporomandibular disorders and their treatment can also be contributing factors to this issue.

The mean attitude score of 3.1 (of the total 5.0) implies that the practicing dentists of Tehran have an appropriate level of attitude towards TMD patients.

With respect to the most qualified specialty for temporomandibular disorders, all experts, 66.7% of specialists, and 38.2% of dentists opted for prosthodontists. Comparing the opinion of experts and that of dentists, it seems that the higher the knowledge about TMD, the greater is the tendency to choose prosthodontics as the most qualified specialty for TMD. Furthermore, the only group of specialists that was thoroughly willing to visit TMD patients for diagnosis and treatment were prosthodontists.

All three groups (consisting of general dental practitioners, specialists and academic experts) reached a consensus regarding the inadequacy of undergraduate dental education in TMD and orofacial pain, which emphasizes the need to develop and strengthen the curriculum regarding the issue.

Several reports and studies have shown a high percentage of the population suffering some form of TMD.,^4 , 8^ Literature yields figures of millions of people having TMD.^13 - 15^ The question arises as to why an effective treatment is not presented to these patients. There are several assumptions running through the mind; in Iran, dentistry is going through a direction where a series of meddling factors have affected the treatment of TMD patients. These factors range from insufficient knowledge, treatment complications, and blurry prognosis to economic problems and patient selection by the dentist. The elective nature of the treatment in the opinion of some of the respondents and probably the high tolerance of these patients are other contributing factors.

## Conclusion

In spite of the high prevalence of TMD, the level of knowledge and attitude of TMD among the assessed group of general dental practitioners is insufficient. The majority of them are not willing to visit TMD patients, believing they did not have sufficient professional education on the subject, nor the diagnosis and treatment of TMD. Hence, curricula of the dental schools and continuous education programs need to be revised to ensure a proper practice in the diagnosis and treatment of TMD patients.
